# Publisher Correction: Unravelling the immune signature of *Plasmodium falciparum* transmission-reducing immunity

**DOI:** 10.1038/s41467-018-03769-w

**Published:** 2018-04-11

**Authors:** Will J. R. Stone, Joseph J. Campo, André Lin Ouédraogo, Lisette Meerstein-Kessel, Isabelle Morlais, Dari Da, Anna Cohuet, Sandrine Nsango, Colin J. Sutherland, Marga van de Vegte-Bolmer, Rianne Siebelink-Stoter, Geert-Jan van Gemert, Wouter Graumans, Kjerstin Lanke, Adam D. Shandling, Jozelyn V. Pablo, Andy A. Teng, Sophie Jones, Roos M. de Jong, Amanda Fabra-García, John Bradley, Will Roeffen, Edwin Lasonder, Giuliana Gremo, Evelin Schwarzer, Chris J. Janse, Susheel K. Singh, Michael Theisen, Phil Felgner, Matthias Marti, Chris Drakeley, Robert Sauerwein, Teun Bousema, Matthijs M. Jore

**Affiliations:** 1 Radboud Institute for Health Sciences, Radboud University Medical Center, PO Box 9101, 6500 HB Nijmegen, The Netherlands; 2 Department of Immunology and Infection, London School of Hygiene and Tropical Medicine, Keppel Street, WC1E 7HT London, UK; 3 Antigen Discovery Inc., 92618 Irvine, CA USA; 4 Institute for Disease Modeling, 3150 139th Ave SE, 98005 Bellevue, WA USA; 5 Organisation de Coordination pour la lutte contre les Endémies en Afrique Centrale, BP 288, Yaoundé, Cameroon; 6 Institut de Recherche pour le Développement, MIVEGEC (IRD, CNRS, Univ. Montpellier), 911 Avenue Agropolis, 34394 Montpellier, France; 7 Institut de Recherche en Sciences de la Santé, 399 Avenue de la Liberté, 01 BP 545 Bobo-Dioulasso, Burkina Faso; 8 Faculty of Medecine and Pharmaceutical Science, PO Box 2701, Douala, Cameroon; 9 Radboud Institute for Molecular Life Sciences, Radboud University Medical Center, PO Box 9101, 6500 HB Nijmegen, The Netherlands; 10 Medical Research Council Tropical Epidemiology Group, London School of Hygiene and Tropical Medicine, Keppel Street, WC1E 7HT London, UK; 11 School of Biomedical and Healthcare Sciences, Plymouth University, Drakes Circus, PL4 8AA Plymouth, UK; 12 Department of Oncology, University of Torino, Via Santena 5bis, 10126 Torino, Italy; 13 Department of Parasitology, Leiden University Medical Center (LUMC), Albinusdreef 2, 2333 ZA Leiden, The Netherlands; 14 Department for Congenital Diseases, Statens Serum Institut, DK 2300 Copenhagen, Denmark; 15 Centre for Medical Parasitology at Department of International Health, Immunology and Microbiology, University of Copenhagen and Department of Infectious Diseases, Copenhagen University Hospital, Rigshospitalet, DK 2200 Copenhagen, Denmark; 16 Department of Medicine, University of California Irvine, 92697 Irvine, CA USA; 17 Department of Immunology and Infectious Diseases, Harvard School of Public Health, 02115 Boston, MA USA; 18 Wellcome Center for Molecular Parasitology, University of Glasgow, G12 8TA Glasgow, UK

Correction to: *Nature Communications* 10.1038/s41467-017-02646-2, published online 8 February 2018.

The original version of this Article contained errors in Fig. 3. In panel a, bars from a chart depicting the percentage of antibody-positive individuals in non-infectious and infectious groups were inadvertently included in place of bars depicting the percentage of infectious individuals, as described in the Article and figure legend. However, the *p* values reported in the Figure and the resulting conclusions were based on the correct dataset. The corrected Fig. 3a now shows the percentage of infectious individuals in antibody-negative and -positive groups, in both the PDF and HTML versions of the Article. The incorrect and correct versions of Fig. 3a are also presented for comparison below as Figure 1.

The HTML version of the Article also omitted a link to Supplementary Data 6. The error has now been fixed and Supplementary Data 6 is available to download.

The incorrect version of Fig. 3a is shown below:

The correct version of Fig. 3a is shown here:

