# Supplementary material for: Unravelling the immune signature of Plasmodium falciparum transmission-reducing immunity
Source: Nat Commun. 2018 Feb 8;9:558. doi: 10.1038/s41467-017-02646-2 (PMC5805765; doi:10.1038/s41467-017-02646-2)
Supplement: Supplementary file 1 — Supplementary Information [file 41467_2017_2646_MOESM1_ESM.pdf]

Supplementary Information

Supplementary Figure 1. Antibody responses to the gametocyte protein microarray, with age data for Burkina Faso only. Responses by age are for individuals from Burkina Faso only, which was the only endemic country with substantial numbers of recruits from all age groups. Antibody breadth is the number of proteins reactive above background in each individual. Antibody magnitude is the log2-transformed average signal intensity (SI) for each microarray target, minus the vehicle SI. This equates to the log2-fold change over background. Responses are shown to each protein target by all individuals within given groups**.** For all box-plots, outliers are shown in black while all data points are shown in grey as a bee-swarm.

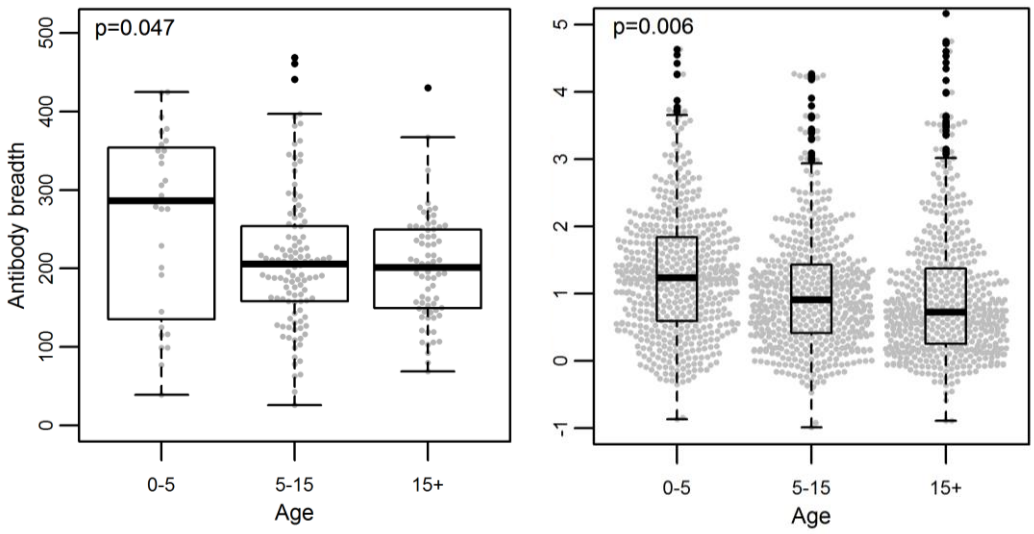

Supplementary Figure 2. Gametocyte density and transmission reducing activity. Gametocyte density and TRA over age (**A** & **B**) are for individual's living in endemic areas only. Gametocyte density is shown to 1000 gametocytes/µL in **A** and **D**. Values above this limit are excluded from the plots (points missing: 0-5=7, 5-15=12, 15+=0), but not the summary statistics for the box-plots (median, IQR). TRA on a continuous scale (**B** & **C**) is presented as absolute TRA for clarity, but the p values are from simple linear regression using log relative infectivity (log[oocysts in treatment mosquitoes/oocysts in control mosquitoes]), to normalise the data and avoid compression at zero. All plots and statistical analysis with age or gametocyte density include only individuals from endemic areas. Grouped TRA (**D**) is presented for individuals with antibodies causing TRA (TRA) ≥90% vs gametocyte positive individuals with antibodies causing TRA <10%, as presented elsewhere. For all box-plots, outliers are shown in black while all data points are shown in grey as a bee-swarm.

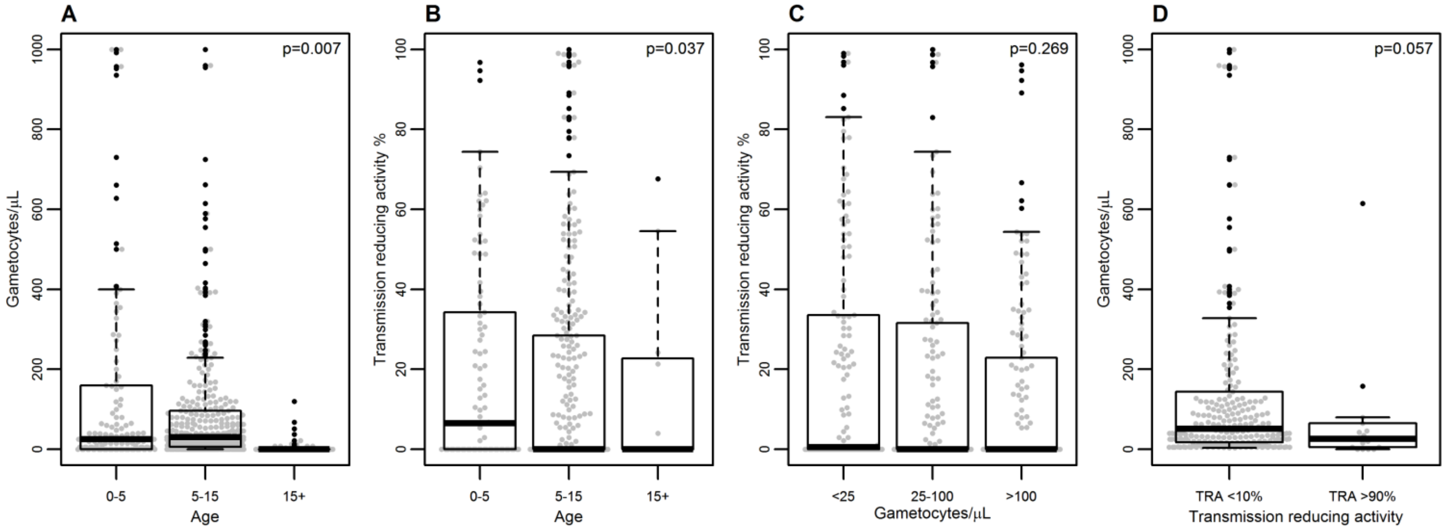

Supplementary Figure 3. Mosquito infection prevalence decreases with breadth of response to novel TRA associated protein across all gametocyte densities. Breadth of response to the 62 novel TRA associated protein targets from the array analysis were binned into quartiles among the population with DMFA data (n=366). Box plots show the median, 25th, 75th 5th and 95th percentiles of mosquito infection prevalence. For all box-plots, outliers are shown as hollow circles.

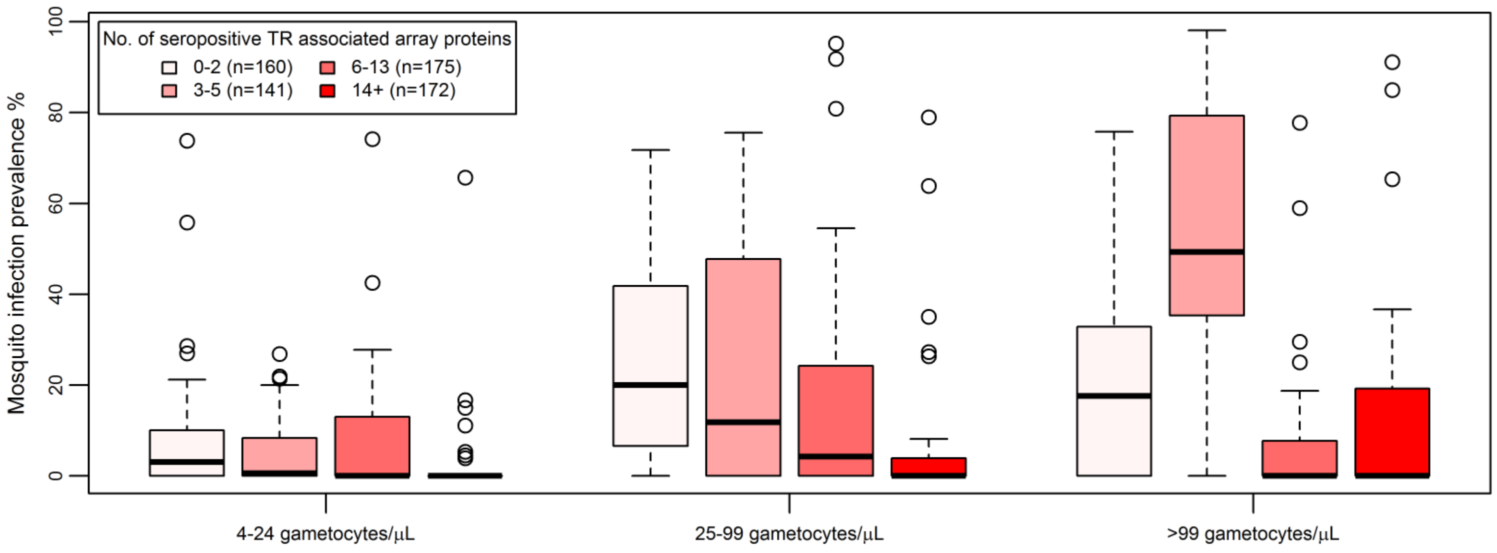

Supplementary Figure 4. TRA of mAbs against Pfs48/45, Pfs230, and novel biomarker PfGEST. For Pfs230 and PfGEST mAB, TRA was calculated against isotypic negative control mAb (*α-CSP*). Pfs48/45 TRA was calculated using human serum as negative control. Details for Pfs48/45 (85RF45.1) mAb production and SMFA is provided in Stone et al. 2014 [1]. Details of Pfs230 mAb production and SMFA are presented in Roeffen et al. 1995 [2]. PfGEST mAb were produced by the Hypothesis-driven Pre-erythrocytic Antigen Target Identification Consortium (HPATIC) and provided by PATH. SMFA was conducted according to previously published protocols [1]. In all mAb based SMFA, the endpoint was oocyst quantification by manual dissection. Between 20 and 60 mosquitoes were dissected in each experiment. P-values are from a generalised linear mixed model (GLMM), as described previously [3].

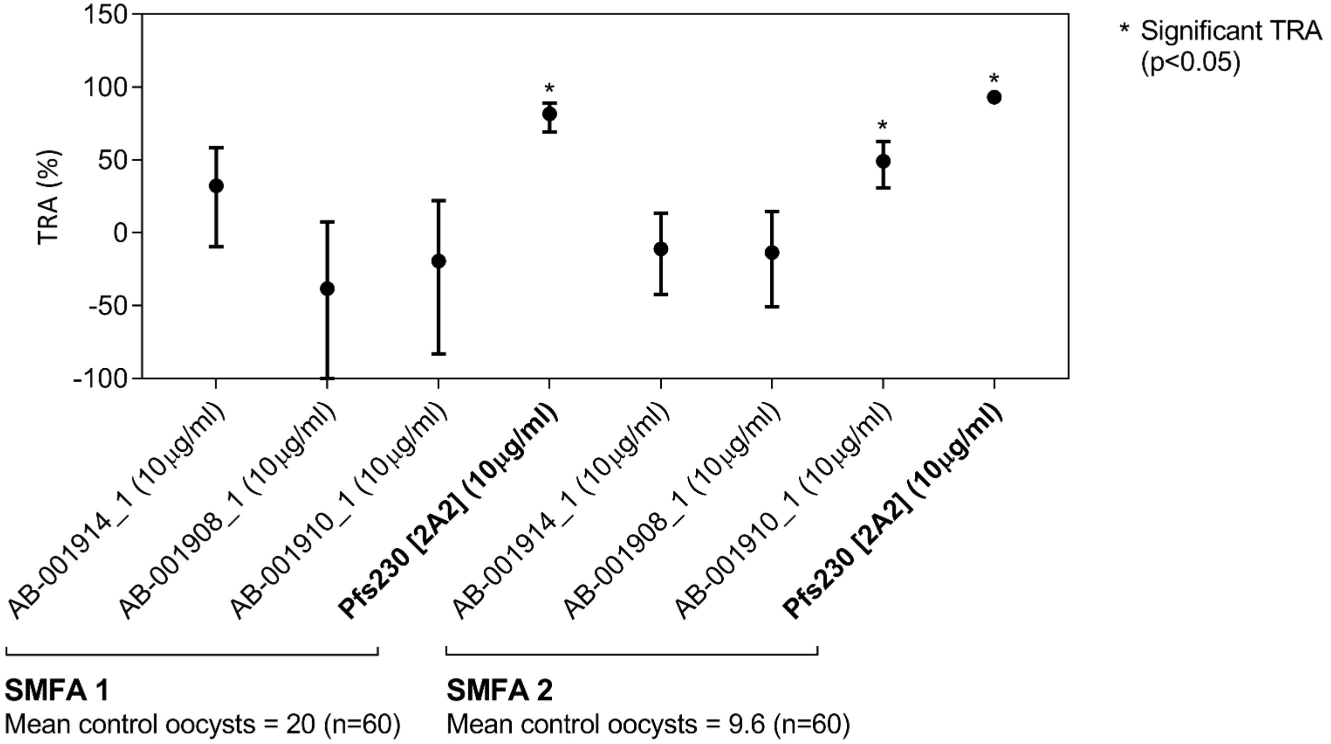

Supplementary Figure 5. Gamete surface immuno-fluorescence assay (SIFA) using wild-type and Pfs48-45 KO NF54 gametes, with PfGEST mAb and IgG/plasma from a malaria exposed serum donor. Donors B and E and naïve donor plasma are described in table 2. SIFA was performed with human antibody sources from whole plasma or total IgG depleted of α-Pfs48/45-10c and α-Pfs230CMB Ab. Δ Pfs48/45 = Pfs48/45 KO [4]. BF = Bright-field, FITC = fluorescein isothiocyanate. GEST mAbs were a mixture of 5μg/mL of all three GEST mAbs described in the methods. Scale bar is 20μm. **A.** Donor sera, **B.** mAb, with serum for comparison.

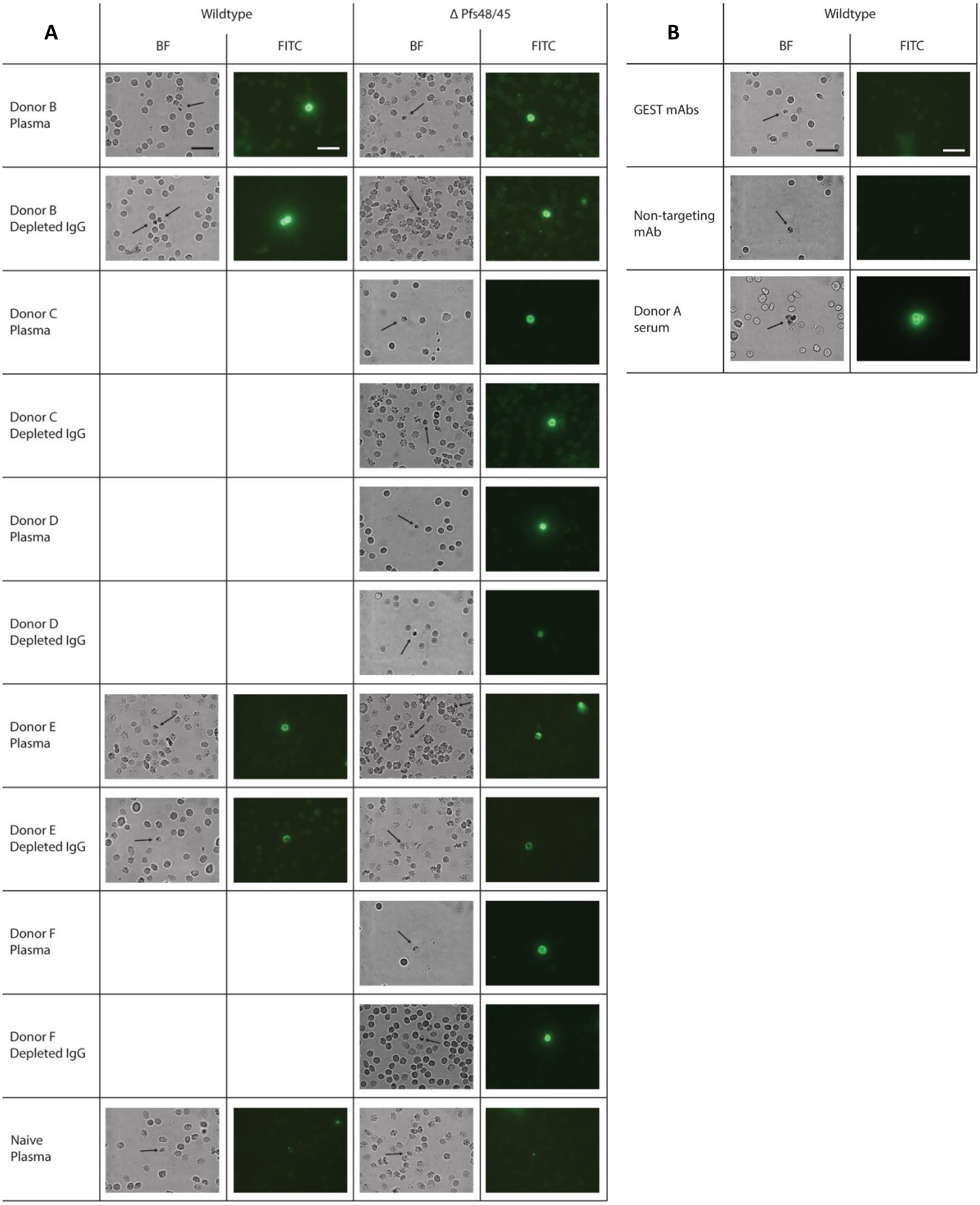

Supplementary Figure 6. Figure 1. Antibody responses to Pfs48/45-10C, Pfs230 and to the microarray proteins with age, with microarray targets that are plausible antibody targets indicated. Antibody responses to conformational, recombinant Pfs48/45 and Pfs230 were measured using ELISA, with antibody intensity given as the ELISA optical density (OD) values (450nm), which is normalised between assay plates by adjustment relative to a consistent point in the linear portion of a standard curve of serially diluted highly reactive human sera. Antibody responses to microarray proteins are given as the log_2-transformed signal intensity (SI) minus the vehicle SI, which equates to the log_2-fold change over this background. All graphs show only individuals from endemic areas (Dutch migrants excluded). Sample size: 0-5 = 131, 5-15 = 366, 15+ = 71. **A & C:** Bars show the seroprevalence of α-Pfs48/45-10C and α-Pfs230 antibodies with age, with Clopper-Pearson confidence intervals. **B & D:** Box plots showing α-Pfs48/45-10C and α-Pfs230 antibody intensity with age. **E**: Box-plots showing responses to the microarray proteins (n=528), and the targets which were identified as having characteristics which make surface expression or involvement in gamete viability possible (n=16). As indicated in the text, these 16 targets represent fragments of 13 unique proteins. Antibody breadth is the number of proteins reactive above background in each individual, within the given groups. **F:** Magnitude of antibody response to microarray proteins and plausible Ab targets. Each spot represents the average SI of response to each protein target by all individuals within given groups. P-values for prevalence data are from likelihood ratio test for differences in seroprevalence between all age groups, derived from logistic regression, and adjusted for gametocyte density. P values for intensity data and response breadth are from an F test for differences in OD/SI between all age groups, derived from linear regression, and adjusted for gametocyte density, or from students t-test (for magnitude only). For all box-plots, outliers are shown with larger black/red dots while all data points are shown in grey/red as a bee-swarm of small dots.

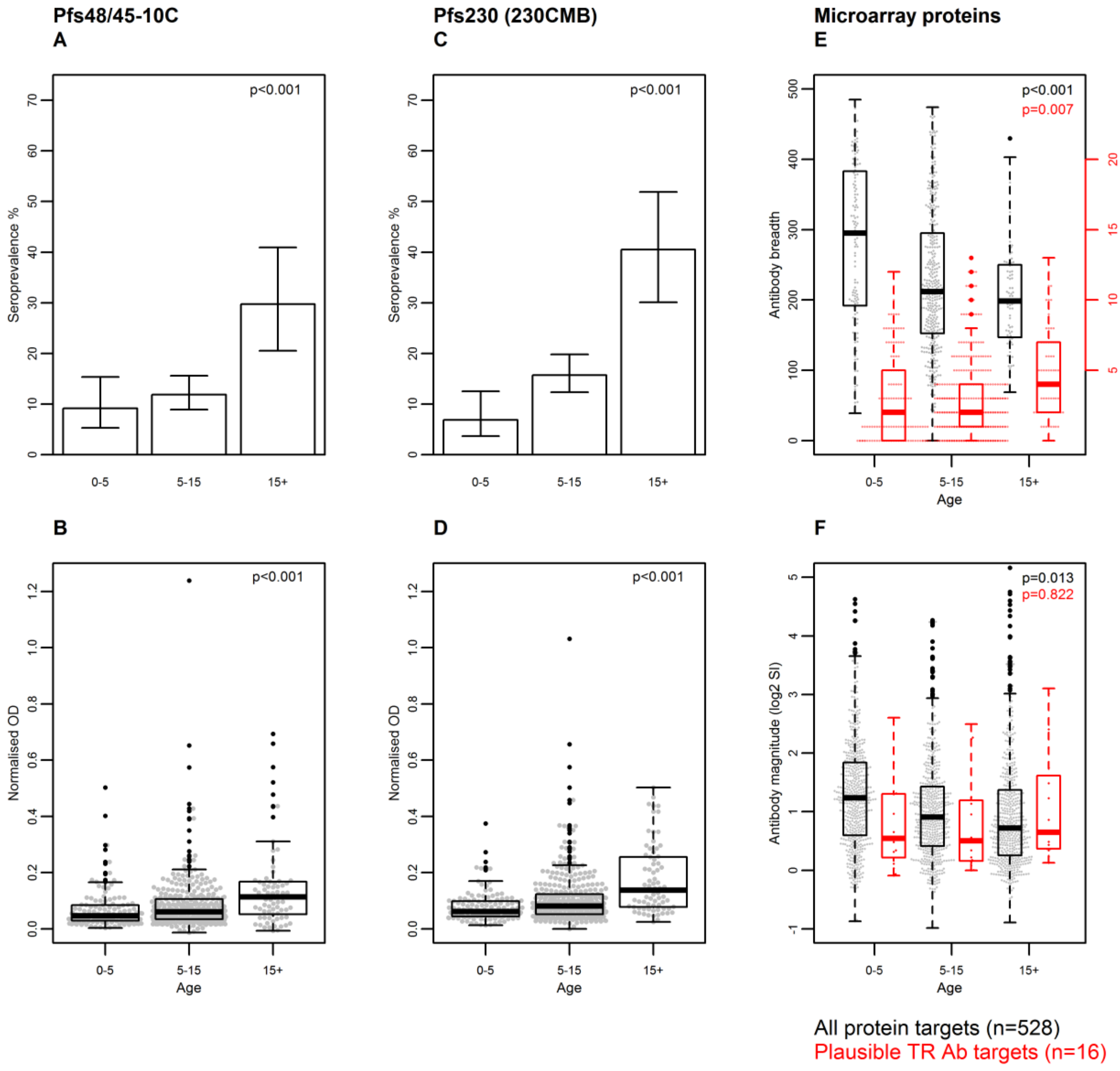

Supplementary Tables

Supplementary Table 1. Transmission-reducing activity and association with Pfs48/45 and Pfs230 antibody responses

**SMFA selection**		**Samples**	**Pfs230 (230CMB)**				**Pfs48/45 (Pfs48/45-10C)**				**Either/both of Pfs230 and Pfs48/45**			
			**% (n/N)**	**OR (TRA >90%)**	**95% CI**	**p-value**	**% (n/N)**	**OR (TRA>90%)**	**95% CI**	**p-value**	**% (n/N)**	**OR (TRA>90%)**	**95% CI**	**p-value**
**Total**	**TRA (all)**	*648*	*16.2% (105/648)*	-	-	-	*14.4% (93/648)*	-	-	-	*21.8% (141/648)*	-	-	-
**Blockers**	**TRA ≥90%**	*22*	*50.0% (11/22)*	-	-	-	*40.9% (9/22)*	-	-	-	*54.5% (12/22)*	-	-	-
**Comparators** all samples	**TRA <90%**	626	15.0% (94/626)	5.8	2.2-15.8	<0.001	13.4% (84/626)	4.0	1.4-11.3	0.009	20.6% (129/626)	5.1	1.9-14.0	0.001
	**TRA <10%**	405	15.6% (63/405)	5.5	2.0-15.3	0.001	14.6% (59/405)	3.7	1.3-10.5	0.015	22.0% (89/405)	4.7	1.7-13.0	0.003
**Comparators** gct positive only	**TRA <90%**	406	12.1% (49/406)	7.3	2.6-20.3	<0.001	11.1% (45/406)	4.7	1.6-13.6	0.004	17.2% (70/406)	6.2	2.2-17.0	<0.001
	**TRA <10%**	254	10.6% (27/254)	8.2	2.9-23.7	<0.001	11.4% (29/254)	4.5	1.5-13.2	0.006	16.9% (43/254)	6.2	2.19-17.45	0.001

% (n/N) Seroprevalence of antibody responses to Pfs230, Pfs48/45, or either protein. n = seropositive individuals, N=total population size

TRA % Transmission-reducing activity of purified IgG in the standard membrane-feeding assay (SMFA), relative to control mosquitoes fed the same gametocyte batch without test antibodies. TRA is the mean of two independent SMFA runs for all samples.

Samples Total number of samples with TRA assessed in the SMFA

n/N Number of samples seropositive in ELISA/Number of samples assessed in the ELISA

OR (TRA ≥90%) Odds ratio of association of seropositivity and TRA, where the presence of TRA is defined as TRA ≥90%, and the absence of TRA is defined by the comparator groups TRA <90%, and <10%. Logistic models are adjusted for gametocyte density.

95% CI 95% confidence intervals of the OR

p-value P-value from the logistic model used to generate the OR

Supplementary Table 2. The TRA of GLURP-R0 compared to dilutions of mAb specific to Pfs48/45 epitope 1 (85RF45.1) and control human serum in the SMFA. Full details of these SMFA experiments are in Stone et al. 2014[5].

Treatment	SMFA 1		SMFA 2		SMFA 3	
	Mean oocysts	TRA %	Mean oocysts	TRA %	Mean oocysts	TRA %
Control (human serum)	6.8	-	13.3	-	17.9	-
85RF45.1 mAb (0.15µg/ml)	6.3	7.2	11.2	15.8	30.7	-71.4
85RF45.1 mAb (0.3µg/ml)	2.9	57.0	13.6	-2.5	16.1	10.2
85RF45.1 mAb (0.6µg/ml)	1.4	79.8	7.5	43.7	9.2	48.7
85RF45.1 mAb (1.25µg/ml)	0.1	98.0	1.2	90.7	1.9	89.4
85RF45.1 mAb (2.5µg/ml)	0.1	99.0	0.4	96.7	0.3	98.5
GLURP mAb (2.5µg/ml)	6.9	-2.2	14.1	-6.0	12.1	32.5

Supplementary References

1 Stone, W. J. *et al.* A scalable assessment of Plasmodium falciparum transmission in the standard membrane feeding assay using transgenic parasites expressing GFP-luciferase. *J Infect Dis*, (2014).

2 Roeffen, W. *et al.* Transmission blockade of Plasmodium falciparum malaria by anti-Pfs230-specific antibodies is isotype dependent. *Infection and Immunity* 63, 467-471, (1995).

3 Churcher, T. S. *et al.* Measuring the blockade of malaria transmission – An analysis of the Standard Membrane Feeding Assay. *Int. J. Parasitol.* 42, 1037-1044, (2012).

4 van Dijk, M. R. *et al.* A Central Role for P48/45 in Malaria Parasite Male Gamete Fertility. *Cell* 104, 153-164, (2001).

5 Stone, W. J. *et al.* A scalable assessment of *Plasmodium falciparum* transmission in the standard membrane-feeding assay, using transgenic parasites expressing green fluorescent protein-luciferase. *J Infect Dis* 210, 1456-1463, (2014).
