# Supplementary material for: Unravelling the immune signature of Plasmodium falciparum transmission-reducing immunity
Source: Nat Commun. 2018 Feb 8;9:558. doi: 10.1038/s41467-017-02646-2 (PMC5805765; doi:10.1038/s41467-017-02646-2)
Supplement: Supplementary file 3 — Description of Additional Supplementary Files [file 41467_2017_2646_MOESM3_ESM.pdf]

File Name: **Supplementary Data 1**
Description: **Full list of proteins included on the protein microarray**

File Name: **Supplementary Data 2**
Description: **Recombinant proteins (Pfs48/45 and Pfs230) or microarray targets (n=62) with TRA associated antibody responses**

File Name: **Supplementary Data 3**
Description: **Empirical Bayes-moderated t-test for average response to microarray protein targets: Comparison of responses by individuals with ≥90% TRA, and gametocyte positive individuals with <10% TRA**

File Name: **Supplementary Data 4**
Description**: Z-scores and seropositivity for Pfs48/45, Pfs230 and TRA associated array targets (n=62), among individuals with high level TRA (>90%; n=22).** Z-scores were calculated as as follows, separately for each protein: (sample log2 SI (signal intensity) – mean log2SI for all individuals)/standard deviation log2SI for all individuals. See key to table for full details.

File Name: **Supplementary Data 5**
Description: **Full list of proteins with TRA associated antibody responses in our array analysis**

File Name: **Supplementary Data 6**
Description: **Protein and peptide proteome for *P. falciparum* schizonts.** To produce a measure of gametocyte specificity using data generated in a single laboratory, gametocyte and asexual (trophozoites and schizonts) stage-specific expression datasets were generated in relative expression values to compare protein abundances (label-free quantitative values) between the stages, which are presented in supplemental data 1 as a fold change value. Gametocyte and trophozoites proteomic data have been previously presented (doi: 10.1074/mcp.M900479-MCP200), but the schizont data are summarized here for easy reference. The full *P. falciparum* Schizont mass spectrometry proteomics data have been deposited to the ProteomeXchange Consortium (http://proteomecentral.proteomexchange.org) via the PRIDE partner repository with the dataset identifier PXD008250.
